# ASpedia-R: a package to retrieve junction-incorporating features and knowledge-based functions of human alternative splicing events

**DOI:** 10.1093/bioadv/vbae071

**Published:** 2024-05-11

**Authors:** Daejin Hyung, Soo Young Cho, Kyubin Lee, Namhee Yu, Sehwa Hong, Charny Park

**Affiliations:** Research Institute, National Cancer Center, Goyang, Gyeonggi-do 10408, Republic of Korea; Department of Molecular & Life Science, Hanyang University, Ansan-si, Gyeonggi-do 15588, Republic of Korea; Department of Biochemistry and Molecular Genetic, University of Virginia, Charlottesville, VA 22908, USA; Research Institute, National Cancer Center, Goyang, Gyeonggi-do 10408, Republic of Korea; Research Institute, National Cancer Center, Goyang, Gyeonggi-do 10408, Republic of Korea; Research Institute, National Cancer Center, Goyang, Gyeonggi-do 10408, Republic of Korea

## Abstract

**Motivation:**

Alternative splicing (AS) is a key regulatory mechanism that confers genetic diversity and phenotypic plasticity of human. The exons and their flanking regions include comprehensive junction-incorporating sequence features like splicing factor-binding sites and protein domains. These elements involve in exon usage and finally contribute to isoform-specific biological functions. Splicing-associated sequence features are involved in the multilayered regulation encompassing DNA and proteins. However, most analysis applications have investigated limited sequence features, like protein domains. It is insufficient to explain the comprehensive cause and effect of exon-specific biological processes.

**Results:**

With the advent of RNA-seq technology, global AS event analysis has deduced more precise results. As accumulating analysis results, it could be a challenge to identify multi-omics sequence features for AS events. Therefore, application to investigate multi-omics sequence features is useful to scan critical evidence. ASpedia-R is an R package to interrogate junction-incorporating sequence features for human genes. Our database collected the heterogeneous profile encompassed from DNA to protein. Additionally, knowledge-based splicing genes were collected using text-mining to test the association with specific pathway terms. Our package retrieves AS events for high-throughput data analysis results via AS event ID converter. Finally, result profile could be visualized and saved to multiple formats: sequence feature result table, genome track figure, protein–protein interaction network, and gene set enrichment test result table. Our package is a convenient tool to understand global regulation mechanisms by splicing.

**Availability and implementation:**

The package source code is freely available to non-commercial users at https://github.com/ncc-bioinfo/ASpedia-R.

## 1 Introduction

Alternative splicing (AS) is a key regulatory mechanism conferring human genetic diversity (Pan *et al.* 2008). Distinct regulatory programs involving splicing have been observed in various tissues and diseases, but they are not yet fully understood. Exons and their flanking regions participate in cis-regulatory splicing, and switch-like exon usage generates multiple isoforms containing distinct functional sequences, such as the protein domain or post-translational modification sites usage (Hyung *et al.* 2018). Mutations or repeats produce unexpected exon. These isoforms regulate distinct biological processes. For example, the vascular endothelial growth factor A isoforms have different protein domains, and play antagonistic roles in pro-angiogenic and anti-angiogenic functions (Harper and Bates 2008). Taken together, multiple junction-incorporating sequences participate in exon skipping or confer functional heterogeneity to the isoforms (Hyung *et al.* 2018). Thus, these sequence features provide powerful information for speculating the evidence for AS.

With the advancement of high-throughput technologies, global AS events can be extracted; however, it is a challenge to determine the functional importance according to exon usage. To the best of our knowledge, current analysis approaches in terms of splicing have provided evidence restricted to protein. DIGGER, AltAnalyze, and DoChaP infer protein–protein or domain–domain interactions at the network level. Meanwhile, MutSpliceDB collects effective splice site variants in AS regions, including the flanking regions (Emig *et al.* 2010, Gal-Oz *et al.* 2021, Louadi *et al.* 2021, Palmisano *et al.* 2021). These approaches have the advantage to find evidence for global splicing regions. However, each application provides only single-layered information such as the protein domain. Therefore, multi-layered sequence collection could be more useful to elucidate the heterogeneous evidence of splicing.

Hence, we developed an application to explore junction-incorporating sequence features that exist in the human AS event regions. The ASpedia database had already been developed, but the retrieval functions were performed through web application (Hyung *et al.* 2018). Therefore, partial functions like customized visualization are uncomfortable for users. The previous database was referred to restricted genome version, hg18 and hg19. Therefore, we updated the database context for the recent genome version. Meanwhile, we added a novel content, knowledge-based splicing gene sets to regulate pathways. Current gene set databases were mostly developed to investigate total gene-level regulation. Therefore, isoform-specific signaling pathways were hard to identify from AS analysis. Here, we interrogated pathways regulated by splicing using text-mining technology to improve the biological function determination for AS genes. Totally, our package embedded splicing-customized retrieval results from the RNA-seq analysis. Furthermore, genome track visualization summarized the sequence features of the genomic coordinates of the spliced exons.

Spliced isoforms confer functional heterogeneity to the genes. However, a systemic investigation is inadequate about pathways regulated by spliced genes. Therefore, we developed a database for knowledge-based spliced gene set elucidating pathways, and the associations were collected through text mining (Lee *et al.* 2023). Our package includes a knowledge-based gene-pathway association database extracted from PubMed (*n* = 63 229). In a previous study, we extracted pathway and splicing gene associations using the Stanford Core Natural Language Processing Parser (Manning *et al.* 2014). Gene names, including synonyms, were obtained from the NCBI database. Pathway terms refer to MSigDB pathway names (curated, ontology, and hallmark) and were recognized by the rule-based approach PathNER for several phrases to have equal meaning (Wu *et al.* 2013, Liberzon *et al.* 2015). Next, the collected pathway–gene associations were validated by a co-occurrence test according to terminology frequency. The co-occurrence test was performed for only pathway–gene associations to exist within a single sentence. The co-occurrent frequency between the pathway and the gene was ranked by merging the *P*-value ranks of the three co-occurrence tests (Lee *et al.* 2023). Finally, we performed a knowledge-based AS gene set enrichment analysis for pathways, considering only gene sets that passed the co-occurrence test and had a reliable gene set size (≥20). When demonstrating our splicing gene sets for several pathways using multiple RNA-seq datasets, our knowledge-based dataset clearly exhibited better performance than other splicing genes derived from differential AS (DAS) analysis in several pathways (Lee *et al.* 2023). Finally, to interpret the biological processes facilitated by AS genes, our package can perform a gene set enrichment test to refer to this knowledge-based pathway-splicing gene set.

Detailed database statistics are summarized in the ASpedia database manual and Supplementary Table S1. The result output generated by our package is composed of 12 features to be 23 columns. These features and columns are descripted in Supplementary Table S2.

## 2 Methods and results

### 2.1 Database development for sequence features and knowledge-based splicing gene–pathway associations

**Figure 1.**
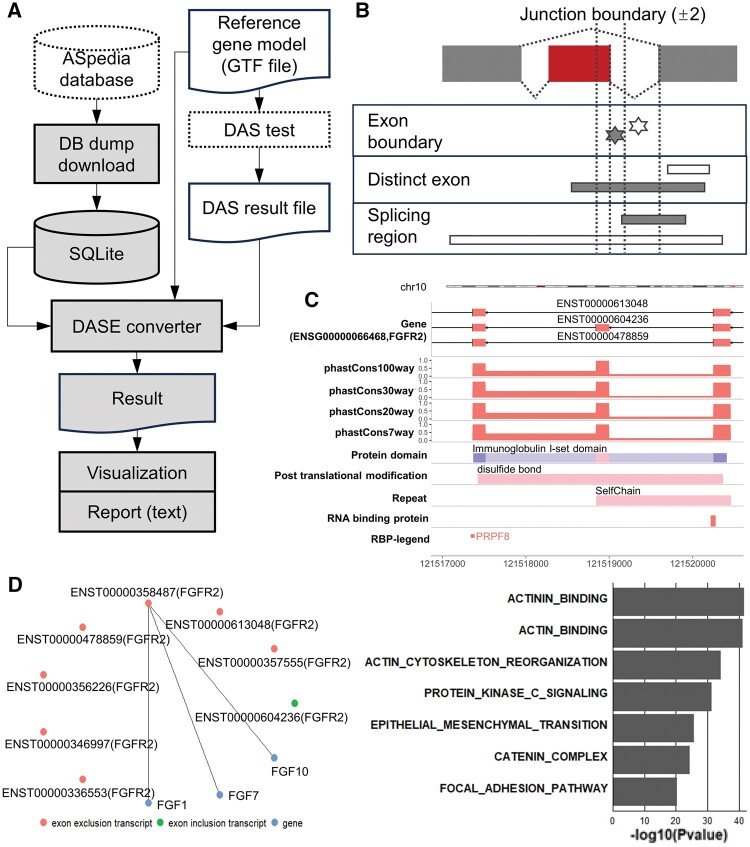
ASpedia-R workflow, and an execution example. (A) The workflow described the analysis process from embedded database establishment to result reporting. Gray objects were internally controlled package processes and databases. (B) Sequence feature extraction strategy presentation to establish our database. Point mutations (gray star) was chosen from distinct exon and its boundary of AS events (first track). Coding region profile-like protein domain was chosen from overlapped with a distinct exon (rectangle; second track). Splicing regions involved in splicing factor-binding sites were selected to be exon or intron specific location except common region of AS events (rectangle; third track). (C) A genome track visualization example for FGFR2 splicing event encompassing gene model of the splicing event and sequence features interrogated from the ASpedia database. (D) Additionally, isoform-specific protein–protein interaction, and AS gene enrichment test results were also supported in our package.

The main components of our database are the sequence features encompassing multi-omics. We primarily collected sequence features around exons and their flanking regions, and the context encompassed DNA (conservation, mutation, and repeat), RNA [miRNA-binding site, non-sense-mediated decay (NMD), RNA-binding proteins, and exon usage multiple samples], and proteins (protein domain, protein–protein interaction, post-modification site, and subcellular localization) (Fig. 1A) (Hyung *et al.* 2018). Sequence features were screened for distinct splicing regions, such as skipped exons or retained introns, except for common AS event regions (Fig. 1B). For example, point mutations were extracted from exon boundary regions and NMD from distinct exon regions. RNA-binding proteins were investigated to locate splicing regions. Additionally, we investigated isoform-specific protein interactions resulting from splicing. Besides the previous version to support five AS types, we have expanded our database to seven AS types alternative acceptor, donor site, skipping exon (SE), mutually exclusive exon, retained intron, and alternative first and last exon. AS genome version was also updated for human genome GRCh38 for gene model ENSEMBL and RefSeq.

Spliced isoforms confer functional heterogeneity to the genes. However, a systemic investigation is inadequate about pathways regulated by spliced genes. Therefore, we developed a database for knowledge-based spliced gene set elucidating pathways, and the associations were collected through text mining (Lee *et al.* 2023). Our package includes a knowledge-based gene–pathway association database extracted from PubMed (*n* = 63 229). In a previous study, we extracted pathway and splicing gene associations using the Stanford Core Natural Language Processing Parser (Manning *et al.* 2014). Gene names, including synonyms, were obtained from the NCBI database. Pathway terms refer to MSigDB pathway names (curated, ontology, and hallmark) and were recognized by the rule-based approach PathNER for several phrases to have equal meaning (Wu *et al.* 2013, Liberzon *et al.* 2015). Next, the collected pathway–gene associations were validated by a co-occurrence test according to terminology frequency. The co-occurrence test was performed for only pathway–gene associations to exist within a single sentence. The co-occurrent frequency between the pathway and the gene was ranked by merging the *P*-value ranks of the three co-occurrence tests (Lee *et al.* 2023). Finally, we performed a knowledge-based AS gene set enrichment analysis for pathways, considering only gene sets that passed the co-occurrence test and had a reliable gene set size (≥20). Finally, to interpret the biological processes facilitated by AS genes, our package can perform a gene set enrichment test to refer to this knowledge-based pathway-splicing gene set.

Detailed database statistics are summarized in the ASpedia database manual and Supplementary Table S1.

### 2.2 Analysis workflow for RNA-seq dataset

Our application provides a retrieval system for exploring multiple AS events by generating own AS IDs, and interrogates all splicing-incorporating profiles around splicing coordinates (Fig. 1A). To allow large-scale AS event retrieval, our package could effectively search for DAS analysis result via data conversion system. First, ASpedia-R constructed a reference database based on SQLite. An AS ID must be generated for each AS event before queries can be generated. Subsequently, the user can query sequence features of AS events from input profile. To conveniently generate AS IDs, our package embedded a converter for several DAS analysis tools (rMATS, SUPPA, and spliceR) (Shen *et al.* 2014, Vitting-Seerup *et al.* 2014, Trincado *et al.* 2018) (Supplementary Fig. S1). In a case of other data formats, the user can easily generate input IDs to follow our package format. Our package allowed us to query exon-level DAS test results using one-to-one AS-key ID mapping. Isoform-level analysis, such as spliceR methods, passes through a two-step conversion: (i) isoform-to-exon coordinates and (ii) exon-to-AS ID keys. Finally, the query results for all sequence features are summarized in a table format. Each AS event was visualized in the genome track format (Fig. 1C). The protein–protein interaction network demonstrated isoform-specific protein interactions (Fig. 1D). Additionally, biological processes derived from DAS genes were investigated by gene set enrichment test from our knowledge-based database. The knowledge-based gene set database followed list object to be equal with gmt format. Therefore, our database could be applicable to other GSEA packages sharing common gmt format gene set reference. This result is summarized in a bar plot. All table results can be saved in the CSV format. An example script to describe workflow could be referred in Supplementary method.

### 2.3 Case studies

To evaluate our package, we analyzed an RNA-seq dataset in triplicate (GSE75489) (Yang *et al.* 2016). The dataset compares wild-type cell lines with the splicing factor epithelial splicing regulatory proteins (ESRP)1/2 knockdown model of H358 cells. RNA-seq dataset were aligned using the genomic data commons pipeline, and the DAS test was performed using rMATS (Shen *et al.* 2014). Finally, we obtained 923 DAS events (*P* < 0.05, |dPSI|>0.1). Among these results, SE exclusion events were dominant, which is consistent with previous ESRP1/2 studies (Yang *et al.* 2016). We investigated the sequence features of the resulting AS events using our application, and 279 DASs were found to contain sequence features. Our result annotated AS events; 26.5% AS events included repeat sequences; 93.9% splicing-inducing point mutations; 84.6% identified-splicing factor-binding sites; 38% protein domain alterations; 58.8% post-translational modification sites; 23.3% isoform-specific interactions; and 8.6% isoform-specific subcellular localization. When testing feature dependency using Fisher’s exact test, protein domain alterations have an association with post-translational modification (*P *=* *0.06, odds ratio = 1.6). We interpreted an AS event example from our result. AS of *BMP1* exon 16 induced the alteration of Calcium-binding EGF domain (protein domain). Additionally, *BMP1* AS events consequently alter cell–cell adhesion (GO BP) by *BMP1* isoform ENST00000520970 interaction with *DMP1* (isoform PPI) (Vadon-Le Goff *et al.*, 2015). Next, to demonstrate consistency with previous studies on ESRP1/2, we performed GSEA to refer to knowledge-based splicing gene sets using our package and database. Input DAS genes nominated pathways: “apical junction” and “epithelial-to-mesenchymal transition” (EMT; adjusted-*P *=* *9.48e-20; Fig. 1D). Our results agree with those of a previous study emphasizing the splicing role of ESRP1 during EMT (Yang *et al.* 2016). Among the protein evidence, we confirmed the sequence features from fibrosis growth factor receptor (*FGFR*)2 exon skipping events (Fig. 1D). Our package visualization highlighted the exon to belong in the “Immunoglobulin I-set” protein domain and the “disulfide bond” post-translational modification site. The identified *FGFR2* SE event can switch from the epithelial to the mesenchymal isoform (Warzecha *et al.* 2009). Additionally, the *FGFR2* exon inclusion isoform interacted with FGF1, and the exclusion isoforms with *FGF10* and *FGF7* (Fig. 1D). Our application extracts isoform-specific interactions through interaction network visualization. In summary, we successfully investigated the biological processes of gene splicing. The sequence features of each AS event provide useful genome-tracking information to focus on exon usage.

### 2.4 Computational performance

ASpedia-R operates in an R language environment and is executable in a general-purpose PC environment. We performed the test using a desktop with 48 Gb of RAM, and an Intel i7-6700 3.40 GHz CPU. First, the embedded database establishment using SQLite required 1–2 min for the GRCh38 ENSEMBL gene model, and the database occupied 706 Mb of storage. This step was performed only once, during the first package execution, and the next analysis was executed by reloading a previously established database. Next, the retrieval time was an average of 1–5 min from the three DAS analysis files of rMATS, SUPPA, and spliceR, including 923–4736 DASs. Visualization and reporting for generating a single result file can be executed within a few seconds.

## 3 Conclusion

Comprehensive sequence features existing in splicing exons could explain the change of biological functions and regulatory mechanisms. However, a systematic investigation is burdensome. Previous studies have investigated restricted profiles such as protein domains according to exon skipping. However, our package is useful for interrogating the comprehensive sequence features of AS events. Besides, execution environment is convenient and lightweight. Massive input could be processed to retrieve the sequence profile for each AS events. The results are presented in multiple forms to promote intuitive understanding. Particularly, GSEA using knowledge-based splicing gene sets could provide the pathway evidence collected from literatures. Our package supports a file converter for three extensively used DAS test applications; however, users can apply ASpedia-R to the results of other DAS applications via input file conversion. We expect that ASpedia-R will be useful for systematically discovering the relevant sequence features of various transcriptome datasets.

## Supplementary Material

vbae071_Supplementary_Data
