# Supplementary material for: ASpedia-R: a package to retrieve junction-incorporating features and knowledge-based functions of human alternative splicing events
Source: Bioinform Adv. 2024 May 11;4(1):vbae071. doi: 10.1093/bioadv/vbae071 (PMC11142624; doi:10.1093/bioadv/vbae071)
Supplement: vbae071_Supplementary_Data [file vbae071_supplementary_data.docx]

**Supplementary Figure S1.** To generate ASpedia-R input file, file conversion strategy. Our converter support three DAS applications: rMATS, SUPPA2, and SpliceR. The converter works a step-wise manner by AS and isoform-level result file. Finally, ASpedia-R referred a prepared input file to explore comprehensive sequence features, and output several forms’ results.


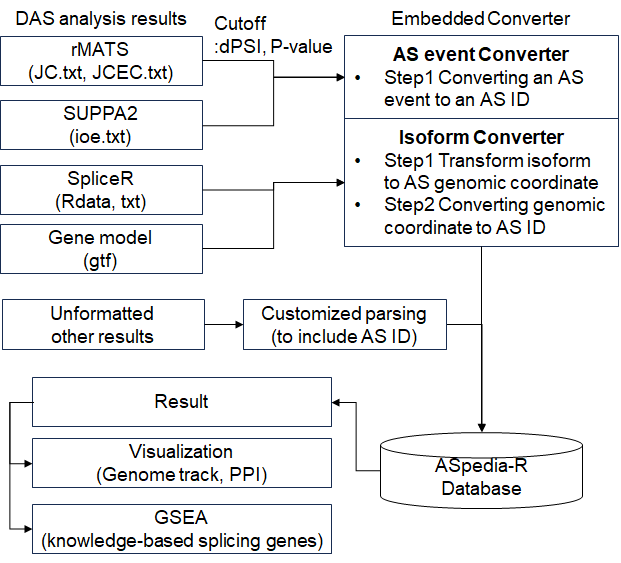


**Supplementary Table S1.** The summary table of ASpedia-R database collection. The number of genes and AS events for each gene model and human reference genome.

| **ENSEMBL** | | | | |
| --- | --- | --- | --- | --- |
|  |  | **hg18** | **hg19** | **GRCh38** |
| **Gene model** | **Total gene** | 33,045 | 55,882 | 40,875 |
|  | **AS gene (AS event)** | 6,663 (16,012) | 13,672 (111,856) | 14,271 (123,989) |
| **ASpedia-R**  **Database** | **NMD** | 293 (574) | 1,238 (4,944) | 1,239 (5,178) |
|  | **miRNA binding site** | 385 (622) | 1,042 (2,894) | 1,067 (3,521) |
|  | **Repeat** | 3,343 (6,482) | 8,880 (46,209) | 10,539 (63,602) |
|  | **Protein domain** | 3,269 (5,845) | 8,701 (37,511) | 8,835 (40,111) |
|  | **PTM** | 4,036 (8,133) | 9,719 (53,130) | 9,933 (56,350) |
|  | **Protein interaction** | 1,358 (2,845) | 2,145 (9,122) | 2,017 (8,719) |
|  | **Subcellular localization** | 373 (925) | 668 (3,662) | 699 (3,928) |
|  | **Transmembrane** | 462 (823) | 1,485 (5,220) | 1,530 (5,800) |
| **GENCODE** | | | | |
|  |  | **hg18** | **hg19** | **GRCh38** |
| **Gene model** | **Total gene** | 42,806 | 62,296 | 63,241 |
|  | **AS gene (AS event)** | 9,981 (60,638) | 13,672 (111,948) | 14,271 (124,082) |
| **ASpedia-R**  **Database** | **NMD** | 734 (2,612) | 1,238 (4,944) | 1,239 (5,178) |
|  | **miRNA binding site** | 683 (1,733) | 1,042 (2,984) | 1,067 (3,521) |
|  | **Repeat** | 6,247 (27,724) | 8,880 (46,272) | 10,539 (63,665) |
|  | **Protein domain** | 5,273 (18,185) | 8,695 (37,519) | 8,830 (40,121) |
|  | **PTM** | 6,714 (28,823) | 9,719 (53,117) | 9,933 (56,397) |
|  | **Protein interaction** | 1,819 (6,052) | 2,145 (9,122) | 2,017 (8,729) |
|  | **Subcellular localization** | 517 (2,169) | 668 (3,662) | 699 (3,935) |
|  | **Transmembrane** | 893 (2,819) | 1,485 (5,220) | 1,530 (5,801) |
| **RefSeq** | | | | |
|  |  | **hg18** | **hg19** | **GRCh38** |
| **Gene model** | **Total gene** | 28,188 | 48,705 | 67,127 |
|  | **AS gene (AS event)** | 2,948 (5,413) | 10,058 (31,423) | 16,797 (119,511) |
| **ASpedia-R**  **Database** | **NMD** | 100 (144) | 369 (600) | 756 (2,961) |
|  | **miRNA binding site** | 69 (89) | 373 (534) | 1,067 (2,808) |
|  | **Repeat** | 1,306 (2,053) | 4,612 (10,041) | 12,524 (65,511) |
|  | **Protein domain** | 1,159 (1,825) | 5,064 (11,863) | 8,485 (33,153) |
|  | **PTM** | 2,115 (3,394) | 7,935 (21,308) | 11,739 (61,853) |
|  | **Protein interaction** | 1,049 (1,685) | 2,037 (5,085) | 2,247 (9,469) |
|  | **Subcellular localization** | 316 (694) | 781 (2,553) | 902 (4,626) |
|  | **Transmembrane** | 171 (222) | 807 (1,567) | 1,317 (4,287) |

**Supplementary Table S2**

| **Category** | **Colume name** | **Description of values** | **Example** |
| --- | --- | --- | --- |
| Gene profile | gene_symbol | HGNC approved gene symbol. | TP73 |
|  | chr | Chromosome. | chr1 |
|  | as_id | Alternative splicing ID. | chr1:3645891:3646012:3647491:3647629:3648027:3648120 |
|  | as_description_id | Renamed alternative splicing ID. | SE: Involving multiple isoforms |
|  | as_type | Alternative splicing type. | SE |
|  | strand | Strand orientation of genomic coordinates. | + |
|  | gene_name | HGNC approved name for the gene. | tumor protein p73 |
|  | locus_group | A group name for a set of related locus types as defined by the HGNC. | protein-coding gene |
|  | location | Cytogenetic location of the gene. | 1p36.32 |
|  | gene_id | REFSEQ or ENSEMBL gene ID. | ENSG00000078900 |
|  | transcript_id | REFSEQ or ENSEMBL transcript ID. | ENST00000346387,ENST00000604479, |
|  |  |  | ENST00000378280,ENST00000604566 |
|  | exon_inclusion_transcript_id | Transcript id included by this alternative splicing. | ENST00000346387,ENST00000604479 |
|  | exon_exclusion_transcript_id | Transcript id excluded by this alternative splicing. | ENST00000378280,ENST00000604566 |
|  | GO_BP | Gene Ontology terms describing pathways and processes of given gene symbol. | APOPTOSIS GO;POSITIVE REGULATION OF TRANSCRIPTION |
|  | GO_CC | Gene Ontology terms describing localization of given gene symbol. | MITOCHONDRION;NUCLEUS |
|  | GO_MF | Gene Ontology terms describing molecular activity of given gene symbol. | DNA BINDING;TRANSCRIPTION FACTOR ACTIVITY |
| Conservation | conservation_score | Average conservation scores of exonic and intronic regions for this AS region. Conservation scores are reported by each database, exon and intron information is in parentheses. | hg19.100way.phastCons:(E)0.304/(I)0.041; |
|  |  |  | phastCons46way.placentalMammals:(E)0.234/(I)0.045; |
|  |  |  | phastCons46way.primates:(E)0.253/(I)0.072; |
|  |  |  | phastCons46way.vertebrate:(E)0.326/(I)0.041 |
| Variant | dbSNP_variant | Variants (point mutations) of splicing site in dbSNP for this AS regions. dbSNP IDs, position of point mutations, refs, and alts are reported. | rs368114063,chr17:76212746,G>A; |
|  |  |  | rs67347443,chr17:76221717,C>CT |
|  | COSMIC_variant | Variants (point mutations) of splicing site in COSMIC for this AS regions. Position of point mutations, refs, and alts are reported. | chr6:3646012,CG>C;chr6:3646013,G>A |
|  | SPIDEX_variant | Variants (point mutations) of splicing site in SPIDEX for this AS region. Position of point mutations, refs, and alts are reported. | chr4:3645891,G>A/C/T;chr4:3645892,T>A/C/G |
| miRNA | miRNA_binding_site | miRNA binding sites of 3’ UTR region predicted by TargetScan for this AS region. miRNA binding regions and miRNA IDs are reported. | chr10:2038702-2038709,miR-125/351; |
|  |  |  | chr5:2038405-2038413,miR-153 |
| Repeat | repeat | Overlapping repeats regions with this AS region. Repeat database names and repeat regions are reported. And only RepeatMasker is reported repeat class information. | RepeatMasker,SINE,chr17:304973-305104; |
|  |  |  | Simple Repeats,chr3:305848-305880 |
| NMD | NMD | NMD sites in known stop codons for this AS region. Chromosomes and NMD sites are reported. | chr12:120636530;chr12:120636541 |
|  | COSMIC_NMD | NMD sites in novel variant stop codons inferring from COSMIC for this AS region. Chromosomes and NMD sites are reported. | chr10:103344469;chr10:103344504 |
|  | dbSNP_NMD | NMD sites in novel variant stop codons inferring from dbSNP for this AS region. Chromosomes and NMD sites are reported. | chr10:70644615;chr10:70645026 |
| Protein domain | protein_domain | Protein domain using Pfam for this AS region. Pfam domain ID, Pfam domain name, and genomic region are reported. And if the information of proteomic region is available, also reported. | PF07647,SAM domain (Sterile alpha motif), |
|  |  |  | 415-478,chr8:3647601-3649379 |
| Post-translational modification (PTM) | protein_translational_modification | PTM sites collected from PhosphositePlus for this AS region. PTM types, proteomic regions, and genomic regions are reported. | Chain,p310-1400,chr2:49924743-49940115; |
|  |  |  | Phosphorylation site,AA1056-1056,chr11:49932703-49932705 |
| RBP | RBP_splicing_factor | Summary of RBP around alternative splicing regions, and peak detection p-values. Target proteins, p-values, peak genomic region, and peak length are reported. | HNRNPU,6.533499884e-07,chr15:3649426-3649497,72; |
|  |  |  | SF3B1,1.022357417e-08,chr1:3647540-3647574,35 |
| Tissue specific alternative splicing | tissue_as | Tissue specific PSI values using rMATS for this AS region. Tissues and psi values are reported. And only tissues with \|psi-value\| >= 0.05 are reported. | brain:-0.638;thyroid:0.362 |
| Protein interaction (PPI) | isoform_PPI_a | Transcript IDs in this isoform. | ENST00000378288;ENST00000378295 |
|  | isoform_PPI_b | Protein interaction partners with isoform_PPI_a. | ITCH/TP73/NEDD4/UBC; |
|  |  |  | WWOX/HMGB1/PIN1/MAGEA2B/E6/TP73/YAP1 |
| Subcellular localization | isoform_subcellular_localization_id | Transcript IDs in this isoform. | Q9HBH9-1;Q9HBH9-2 |
|  | isoform_subcellular_localization | Isoform specific Subcellular localizations. | Cytoplasm;Nucleus > PML body |

**Supplementary Method**

An example script to execute ASpedia-R proceeds from reading rMATS result to making figure for splicing-associated database profile.

| # check ASpediaR is installed  installed.package.list <- installed.packages()[, 1]  if(is.element("devtools", installed.package.list) == FALSE) {  install.packages("devtools")  }  if(is.element("ASpediaR", installed.package.list) == FALSE) {  devtools::install_github("ncc-bioinfo/ASpedia-R")  }  library(ASpediaR)  data.dir <- paste0(.libPaths()[1], "/ASpediaR/test_data")  result.dir <- paste0(.libPaths()[1], "/ASpediaR/test_result")  if(file.exists(data.dir) == FALSE) {  dir.create(data.dir)  }  if(file.exists(result.dir) == FALSE) {  dir.create(result.dir)  }  se.file.name <- paste0(data.dir, "/SE.MATS.JC.txt")  se.download.url <- "http://combio.hanyang.ac.kr:8080/aspedia_v2/data/ util_test_data/rMATs_ESRP/SE.MATS.JC.txt"  if(file.exists(se.file.name) == FALSE) {  download.file(se.download.url, se.file.name, method="auto")  }  # rMATs result convert to ASpedia-R format  se.result <- asr_converter(se.file.name, program="rMATs", pvalue.cutoff=0.05, dpsi.cutoff=0.1, as.type="SE")  # merge converting result  # rmats.result <- rbind(a3ss.result, a5ss.result, se.result, mxe.result, ri.result)  rmats.result <- se.result  # annotation  annotation.result.dir <- paste0(result.dir, "/annotation_result")  if(file.exists(annotation.result.dir) == FALSE) {  dir.create(annotation.result.dir)  }  rmats.annotation.result <- asr_annotation(rmats.result, gene.model="Ensembl", genome.version="GRCh38", result.dir= annotation.result.dir)  # ASpedia-R result plot  gtf.file.name <- paste0(data.dir, "/Homo_sapiens.GRCh38.99.rm.mt.add.chr.gtf")  gtf.download.url <- "http://combio.hanyang.ac.kr:8080/aspedia_v2/data/ util_test_data/Homo_sapiens.GRCh38.99.rm.mt.add.chr.gtf"  if(file.exists(gtf.file.name) == FALSE) {  download.file(gtf.download.url, gtf.file.name, method="auto")  }  result.plot.dir <- paste0(result.dir, "/result_plot")  if(file.exists(result.plot.dir) == FALSE) {  dir.create(result.plot.dir)  }  asr_plot(rmats.annotation.result, gtf.file.name=gtf.file.name, gene.model="Ensembl", genome.version="GRCh38", gene.name="FGFR2", result.dir=result.plot.dir)  #GSEA result plot  library(rtracklayer)  gsea.result.dir <- paste0(result.dir, "/gsea_result")  if(file.exists(gsea.result.dir) == FALSE) {  dir.create(gsea.result.dir)  }  gtf.data <- import(gtf.file.name)  reference.gene.list <- unique(gtf.data$gene_name)  rmats.annotation.gene.list <- unique(rmats.annotation.result$gene_symbol)  mining_gsea(rmats.annotation.gene.list, gsea.gene.list=reference.gene.list, result.dir=gsea.result.dir) |
| --- |
